# Emergence of a new designated clade 16 with significant antigenic drift in hemagglutinin gene of H9N2 subtype avian influenza virus in eastern China

**DOI:** 10.1080/22221751.2023.2249558

**Published:** 2023-08-28

**Authors:** Xiyue Wang, Kaituo Liu, Yaqian Guo, Yuru Pei, Xia Chen, Xiaolong Lu, Ruyi Gao, Yu Chen, Min Gu, Jiao Hu, Xiaowen Liu, Shunlin Hu, Xin-an Jiao, Xiufan Liu, Xiaoquan Wang

**Affiliations:** a College of Veterinary Medicine, Yangzhou University, Yangzhou, People’s Republic of China; b Jiangsu Co-innovation Center for Prevention and Control of Important Animal Infectious Diseases and Zoonosis, Yangzhou University, Yangzhou, People’s Republic of China; c Jiangsu Key Laboratory of Zoonosis, Yangzhou, People’s Republic of China; d Joint International Research Laboratory of Agriculture and Agri-Product Safety, The Ministry of Education of China, Yangzhou University, Yangzhou, People’s Republic of China

**Keywords:** H9N2, HA gene, new designated clade, genetic evolution, antigenic

## Abstract

H9N2 avian influenza viruses (AIVs) pose an increasing threat to the poultry industry worldwide and have pandemic potential. Vaccination has been principal prevention strategy to control H9N2 in China since 1998, but vaccine effectiveness is persistently challenged by the emergence of the genetic and/or antigenic variants. Here, we analysed the genetic and antigenic characteristics of H9N2 viruses in China, including 70 HA sequences of H9N2 isolates from poultry, 7358 from online databases during 2010–2020, and 15 from the early reference strains. Bayesian analyses based on hemagglutinin (HA) gene revealed that a new designated clade16 emerged in April 2012, and was prevalent and co-circulated with clade 15 since 2013 in China. Clade 16 viruses exhibited decreased cross-reactivity with those from clade 15. Antigenic Cartography analyses showed represent strains were classified into three antigenic groups named as Group1, Group2 and Group3, and most of the strains in Group 3 (15/17, 88.2%) were from Clade 16 while most of the strains in Group2 (26/29, 89.7%) were from Clade 15. The mean distance between Group 3 and Group 2 was 4.079 (95%CI 3.605–4.554), revealing that major switches to antigenic properties were observed over the emergence of clade 16. Genetic analysis indicated that 11 coevolving amino acid substitutions primarily at antigenic sites were associated with the antigenic differences between clade 15 and clade 16. These data highlight complexities of the genetic evolution and provide a framework for the genetic basis and antigenic characterization of emerging clade 16 of H9N2 subtype avian influenza virus.

## Introduction

H9N2 subtype avian influenza virus (AIV) was first isolated in turkeys in 1966 in United States and then became endemic in many countries in Asia, Eurasian, Middle East, and Africa [1,2]. Since the first H9N2 virus in mainland China was isolated in Guangdong in 1994 [3], it has been prevalent in many provinces [4–7]. According to the surveillance of AIVs from 16 provinces in China during 2014-2016, H9N2 was isolated in 54.1% of apparently healthy chickens [8]. From 2016 to 2019, H9N2 had become the dominant AIV subtype (72.75% in the pure isolates) in both chickens and ducks in China [9]. The widespread prevalence of H9N2 in China requires extensive attention.

H9N2 subtype AIV has become a severe concern in China because of its threat to the poultry industry and public health. On the one hand, the circulation of H9N2 in poultry can cause significant economic losses due to declined egg production, weight loss and susceptibility to co-infection with other pathogens [4]. On the other hand, H9N2 AIVs pose a threat to human health. Since December 2015 to March 2023, a total of 87 cases of human infection with H9N2 AIV have been reported, including two deaths [10]. Additionally, the continued prevalence of H9N2 in poultry presents additional opportunities for contributing internal gene cassettes to other subtypes of AIV and enhancing adaptation to mammals, like H5N1, H7N9, H10N8, H5N6, H10N3 and H3N8 AIVs [11–16].

In China, vaccination is the major method for controlling H9N2 AIVs in poultry. The first H9N2 vaccine was approved in the late 1990s, and the most frequent vaccine strains used now are A/chicken/Shandong/F/98 (F/98), A/chicken/Shandong/6/96 (SD696) and A/chicken/Guangdong/SS/94 (SS) [17]. However, due to the rapid mutations of RNA viruses, AIVs are susceptible to occur antigenic drifts under the immunization pressures associated with widespread and prolonged use of vaccines. Several studies have reported antigenic-related amino acid residues of H9N2 subtype AIVs identified by reverse genetic constructions [18–23], and there are evidences suggest that the antigenicity of H9N2 subtype AIV has changed significantly in recent years [24–26], highlighting the importance of continuous AIV surveillance. Surveillance and studies of the genetic evolution of H9N2 have been carried out in China for a long time [27–29]. Epidemiologic and genetic studies revealed that H9N2 subtype AIVs were divided into the Eurasian and American lineages based on the hemagglutinin (HA) genes. The Eurasian lineage has three distinct sublineages: A/chicken/Beijing/1/94-like (BJ/94-like), A/quail/Hong Kong/G1/97-like (G1-like), and A/duck/Hong Kong/Y439/97 (Y439-like or Korean like) [30]. Studies showed that most of H9N2 strains belonged to BJ/94-like clade, while a few belonged to the G1-like and Y439-like clades in China, and strains belonging to American lineages were rare [31]. Based on the median root-to-tip distance and manually merged, the HA gene of H9N2 subtype AIVs in China from 1994 to 2013 was divided into 16 clades (0∼15) [28]. However, there has been a dearth of systematical analysis of genetic and antigenic characteristic of H9N2 AIVs since 2014.

In this study, we conducted monitoring programs of H9N2 viruses in Eastern China from 2010 to 2020 and obtained 70 HA sequences of H9N2 viruses from poultry. Genetic and antigenic analyses revealed that a new designated clade 16 has undergone major switches to antigenic properties, which emerged in April of 2012 and was co-circulated with clade 15 since 2013, showing antigenic similarities between phylogenetically related strains. This study provided an insight into the genetic basis and antigenic characterization of emerging clade 16 of H9N2 subtype avian influenza virus and revealed complexities of the genetic evolution. Materials and Methods

### Ethics statement

This study was carried out in strict accordance with the recommendation in the Guide for the Care and Use of Laboratory Animals of the Ministry of Science and Technology of the People's Republic of China. The animal study protocols were approved by the Jiangsu Administrative Committee for Laboratory Animals (approval number: SYXK-SU-2021-0027) and complied with the guidelines of Jiangsu laboratory animal welfare and ethics of Jiangsu Administrative Committee of Laboratory Animals.

### Sample Collection and virus isolation

Sample Collection and virus isolationRegular live bird market surveillances were conducted in Eastern China (Jiangsu, Anhui, Shandong and Zhejiang provinces). Throat and cloacal swabs were collected into phosphate-buffered saline (PBS) with antibiotics (penicillin and streptomycin) and stored at −70 °C. Specimens were incubated into 9 d-old specific-pathogen-free (SPF) embryonated chicken eggs. The subtype of positive hemagglutination assay(HA) virus was identified by a hemagglutination inhibition assay(HI) with the standard positive sera of different subtypes of AIV, where 4 log2(HI > = 16) was the minimum positive titre. The H9N2 subtype AIVs were grown in SPF embryonated chicken eggs for obtaining allantoic fluid samples.

### RT-PCR and sequencing

Viral RNA was extracted from allantoic fluid with EasyPure Viral DNA/RNA Kit (Trans, Beijing, China). Viral RNA was reverse transcribed to cDNA with a 12 bp primer (5’-AGCAAAAGCAGG-3’). PCR was performed in a 50 μl reaction mixture using specific primers. The forward primer was 5’-AGCGAAAGCAGGGG-3,’ and the reverse primer was 5'- AGTAGAAACAAGGGTGTTTT-3.’ PCR products were purified from 1% (w/v) agarose gel after electrophoresis using DNA Gel Extraction Kit (Axygen, China) and sequenced by the Nanjing GenScript Biotech Co., Ltd. 70 HA sequences of H9N2 strains were sequenced and uploaded to Global Initiative on Sharing All Influenza Data (GISAID) and summarized in the Supplementary Materials (Supplementary Table S1).

### Sequence collection and cleaning

The published sequences of H9N2 sampled from 2010 to 2020 were downloaded from the online databases(downloaded in December 2022) of the NCBI Influenza Research Database [32] and GISAID Database. To obtain a high-quality dataset: (1) Sequences were aligned using MAFFT (v7.3) [33] and trimmed manually in MEGA X [34] to obtain the open reading frames (ORFs). (2) Sequences covering less than 95% completeness of the ORFs and low-quality sequences (sequences that contain 80 or more ambiguous bases and early stop codons) were removed. (3) Sequences online were down-sampled by BioAider (v1.334) [35] with a 98% (cd98 dataset) threshold to minimize the computation resource consumption. Reference sequences were added from Li [28], including early isolated viruses and vaccine strains (Supplementary Table S2).

### Bayesian phylogenetic analysis and clade classification update

The phylogenetic tree of HA was constructed using Bayesian Evolutionary Analysis Sampling Trees (BEAST, v1.10.4) [36] to better understand the evolutionary dynamics of H9N2 subtype AIVs. Missing collection dates of selected sequences were designated to the midpoint of the time interval to confirm that this dataset showed a strong temporal signal by combining phylogenetic inference by IQ-tree(v1.6.12) [37] with root-to-tip genetic distances against time regression by TreeTime(v0.7.5) [38]. Then Strains indicated outliers were removed. The XML files were employed with a (GTR + F + G4) substitution model, an uncorrelated relaxed molecular clock model, and a constant tree prior. The Bayesian Markov Chain Monte Carlo (MCMC) chain lengths were set to 200 million generations with samples for every 20,000 steps to achieve convergence. Then, the log files were assessed by Tracer (v1.7.1) with the effective sample size (ESS) values greater than 200 were required. Lastly, the maximum clade credibility (MCC) trees with median node heights were generated after a 10% burn-in by TreeAnnotator (v1.10.4). The MCC trees were visualized in ggtree [39]. The MCC trees were partitioned into clades with the genetic distance threshold of 0.2 using the Phylopart (v2.1) [40]. Clades were then manually divided into 16 clades according to reported classification [28]. Bar plot was drawn then to study the proportion of isolated strains from clade15 and clade16.

### Hemagglutination inhibition (HI) assay and antigenic cartography

The HI assay was performed to study the antigenic variation between strains. Viruses were inactivated by 0.1% Beta Propiolactone (BPL, Sigma, St. Louis, MO). All inactivated virus preparations were emulsified in mineral oil adjuvant to prepare w/o (water-in-oil) emulsion. To generate the antisera against each H9N2 virus, 4-week-old SPF chickens were inoculated with 0.5 ml of inactivated oil-emulsified vaccine derived from the selected viruses. The HI assay was performed with 1% chicken erythrocytes following the procedures according to the standard protocol described in the OIE Terrestrial Manual. The results of multiple experiments were combined with the geometric mean. To further explore the antigenic differences among the two clades, antigenic map was generated by using the Racmacs package for R [41], which transformed the HI titre to form a 2-dimensional MDS map measured in antigenic units (AU), individually. An antigenic distance of 1 AU is equivalent to a 2-fold dilution in the HI assay, and 2 AU is generally considered to be a significant difference [26]. The mean distance between each group were calculated along with 95% CI (confidence interval).

### Potential antigenic-related amino acid residues analysis

Considering that viruses from the same antigenic groups were phylogenetically closely related, the different amino acid residues in HA1 between clade15 and clade16 were analyzed, which were considered to be the potential antigenic-related amino acid resides. The max proportion of different amino acids in different sites was calculated using mutation analysis of BioAider (v1.334) [35]. The sites with a maximum proportion of less than 70% were displayed in the heatmap and stacked bar chart.

## Result

### Phylogenetic analysis and clade classification update of Hemagglutinin

**Figure 1.**
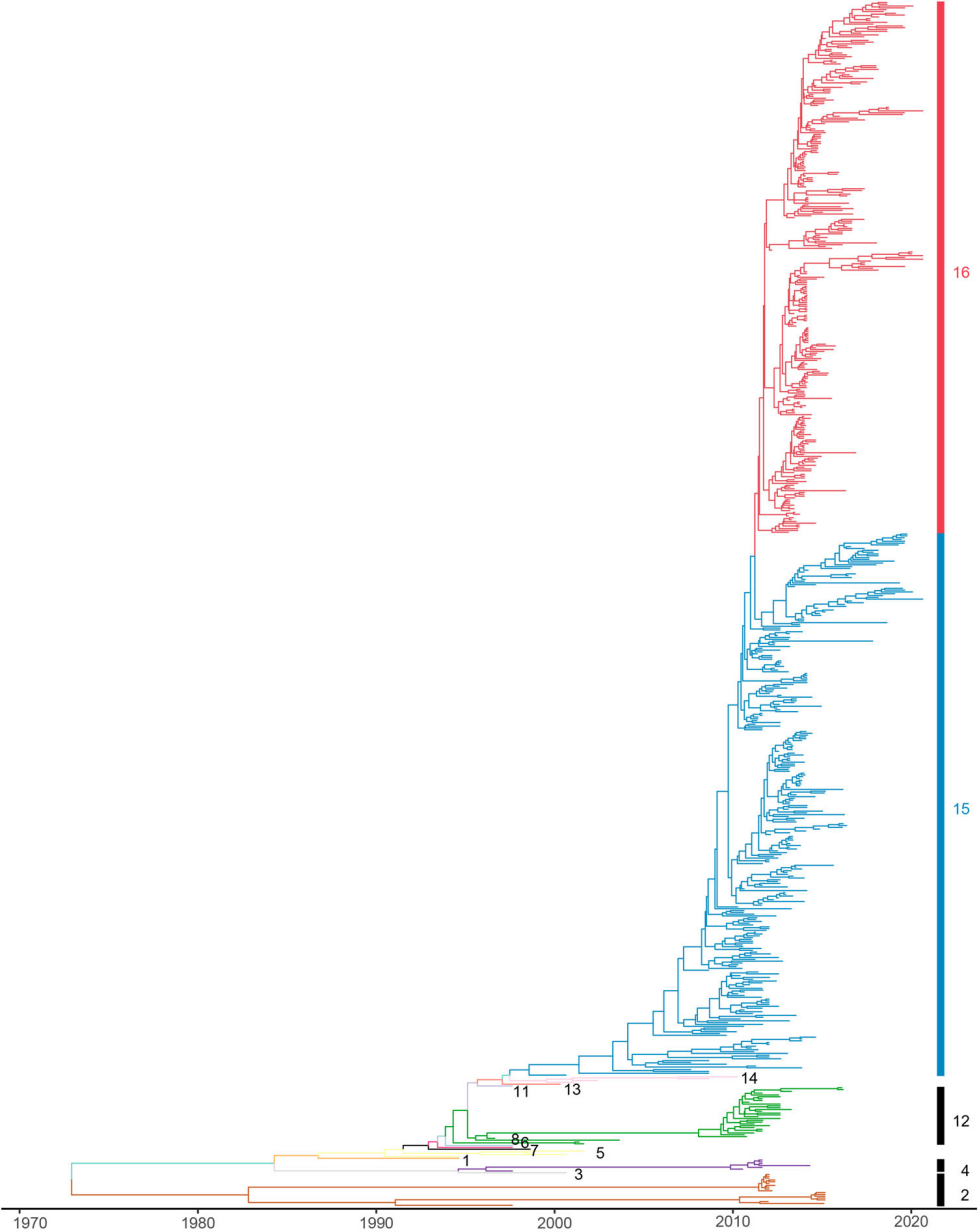
Phylogenetic analysis of HA genes of H9N2 subtype AIV from 2010 to 2020 in China. The maximum clade credibility (MCC) tree of HA gene of H9N2 subtype AIVs from 2010 to 2020 in China. Phylogenetic tree with tip names is depicted in Supplementary Figure S1. Detailed information and clades of viruses in the constructed phylogenetic trees are listed in Supplementary Table S2. Vertical lines with a number indicate the clade partitioned in this study. Different colours represented different clades.

**Figure 2.**
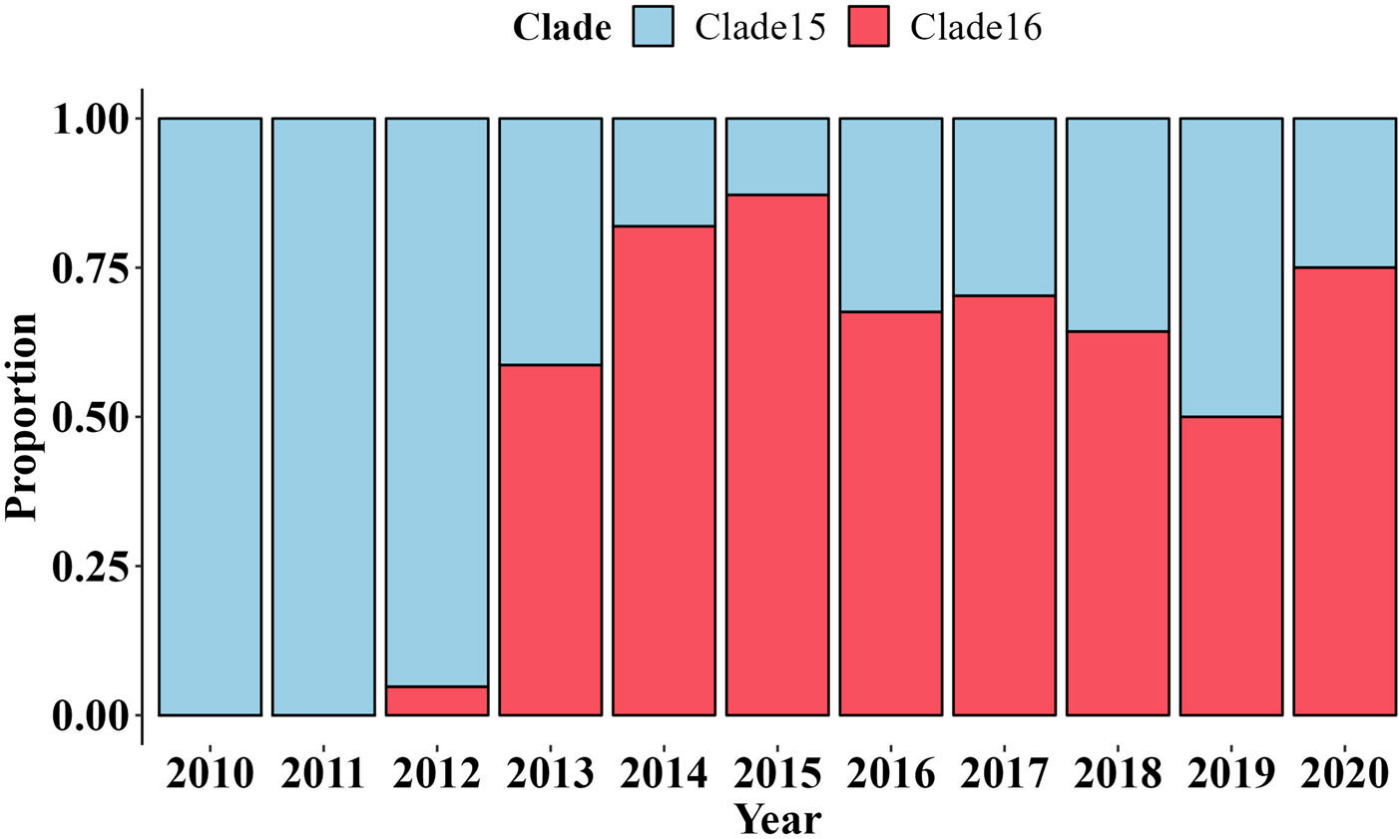
The bar plot indicated the proportion of H9N2 strains from clade15 and clade16 used in this study by year, respectively. The proportion of isolated strains from clade15 was coloured in blue, and the proportion of isolated strains from clade16 in red.

Phylogenetic analysis and clade classification update of Hemagglutinin. To analyze the genetic evolution of the HA genes of H9N2 subtype AIVs, 666 representative isolates were selected, including 70 representative isolates from epidemiological investigation in Eastern China (Supplementary Table S1). The evolution of the temporal signal based on the cd98 HA dataset displayed a temporal signal with R^2^ was 0.89, indicating that the sampling dates could be used for molecular clock calibration for further analysis. The MCC tree indicated that a novel clade of evolution appears, which we named clade 16 (Figure 1, Figure S1, Supplementary Table S2). In detail, it was inferred that clade 16 emerged in April of 2012 according to the most recent common ancestor (tMRCA). The bar plot of the proportion of isolated strains from clade15 and clade16 (Figure 2, Supplementary Table S3) showed that clade16 co-circulate with clade15 after 2013, and gradually displaced clade15, which corresponds to the G57 genotype [28], as the most prevalent clade, after 2013.

### Antigenic drift associated with new genetic clade

**Figure 3.**
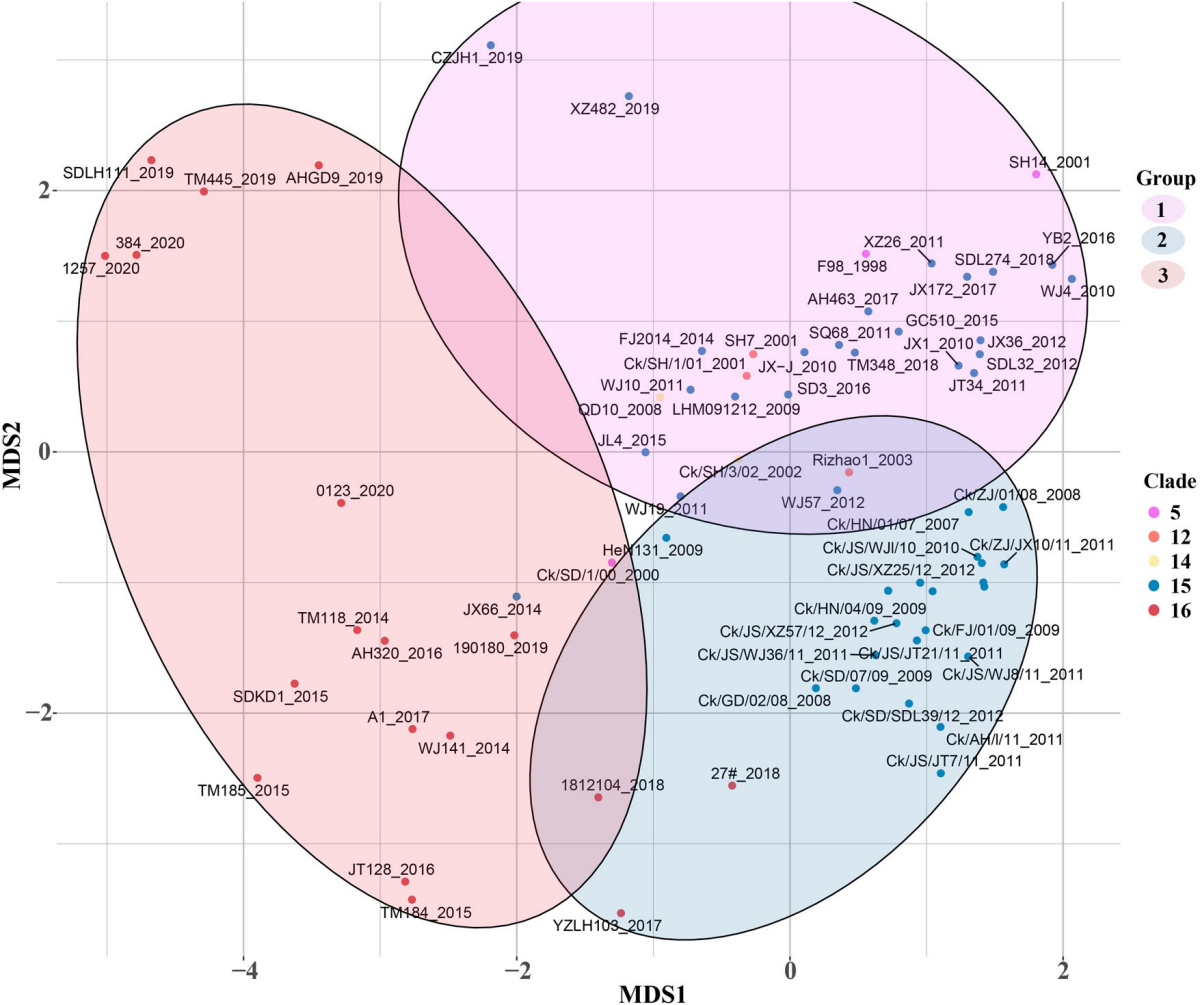
The antigenic cartography of represented H9N2 strains. The represented strains, which were selected in different clades from 1998 to 2020, were coloured differently according to clades and labelled with isolation year and virus name. Clade5 was represented by purple. Clade12 was represented by orange. Clade14 was represented by yellow. Clade15 and Clade16 were represented by blue and red, respectively. The spacing of the grid lines of y axis was equivalent to antigenic unit distance, which corresponds to a two-fold HI difference. All represented strains were divided into 3 groups based on the K-means clustering algorithm and enriched in an oval coloured differently according to antigenic groups.

Seventy-five isolated H9N2 viruses mostly from clade15 and clade16 were selected for antigenic analysis based on the HI tests (Supplementary Table S4). According to the K-means clustering algorithm, the strains were classified into three antigenic groups based on their coordinates in the 2-dimensional MDS coordinate system; taking the collection date of strains in each group into consideration, the three antigenic groups were named as Group1, Group2 and Group3 (Figure 3, Supplementary Table S5). Vaccine strains F/98 and WJ57 were used as representative viruses of antigenic Group 1 and Group 2, respectively. The mean value for the distance between Group 1 and Group 2 was 2.712 (95%CI 2.326–3.100), while the mean distance between Group 3 and Group 1 was 4.427 (95%CI 4.008–4.846) and between Group 3 and Group 2 was 4.079 (95%CI 3.605–4.554), indicating that strains divided into Group 3 had undergone an antigenic drift. Most of the strains in Group 3 (15/17, 88.2%) were from Clade 16 while most of the strains in Group2 (26/29, 89.7%) were from Clade 15, which revealed the new genetic clade was significantly different in antigenic than other clade, including Clade 15, the most prevalent clade before.

### Potential molecular determinants associated with antigen drift

**Figure 4.**
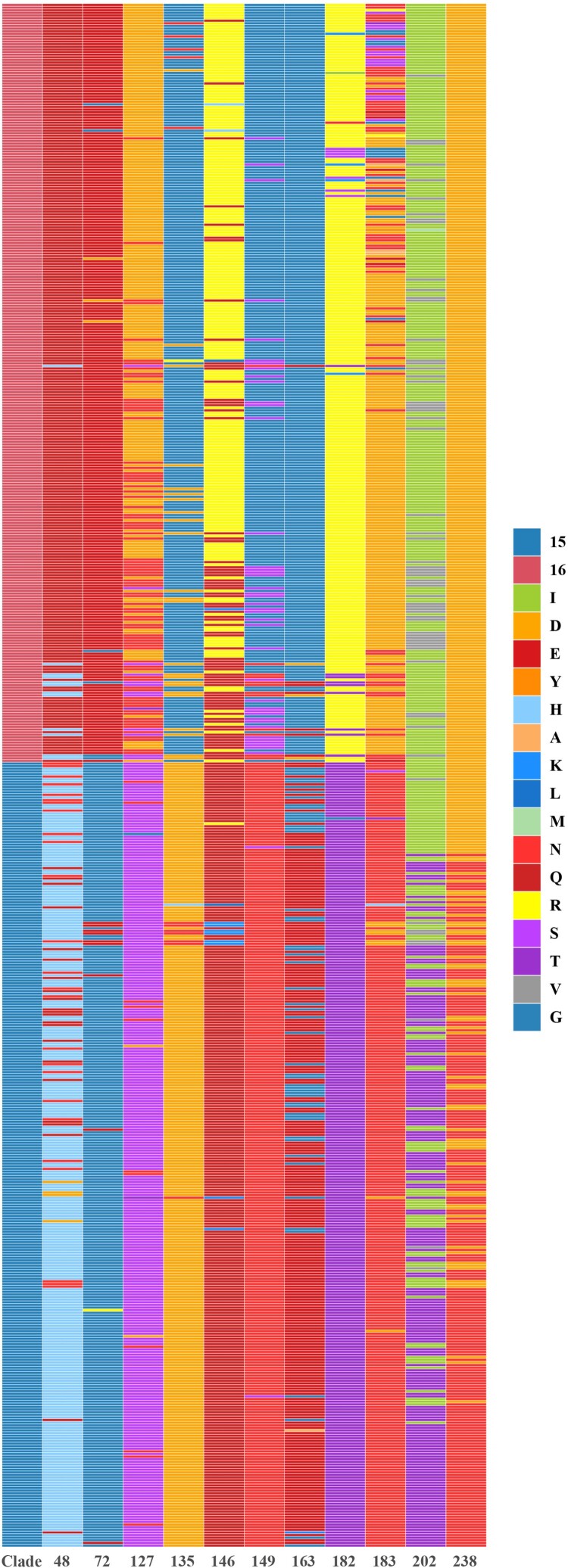
Different amino acid sites in HA1 between clade15 and clade16. The first column represents the clade of each strain, where clade15 was coloured in blue and the clade16 was coloured in red. Other columns represented different amino acid identity with different colours in each site.

**Figure 5.**
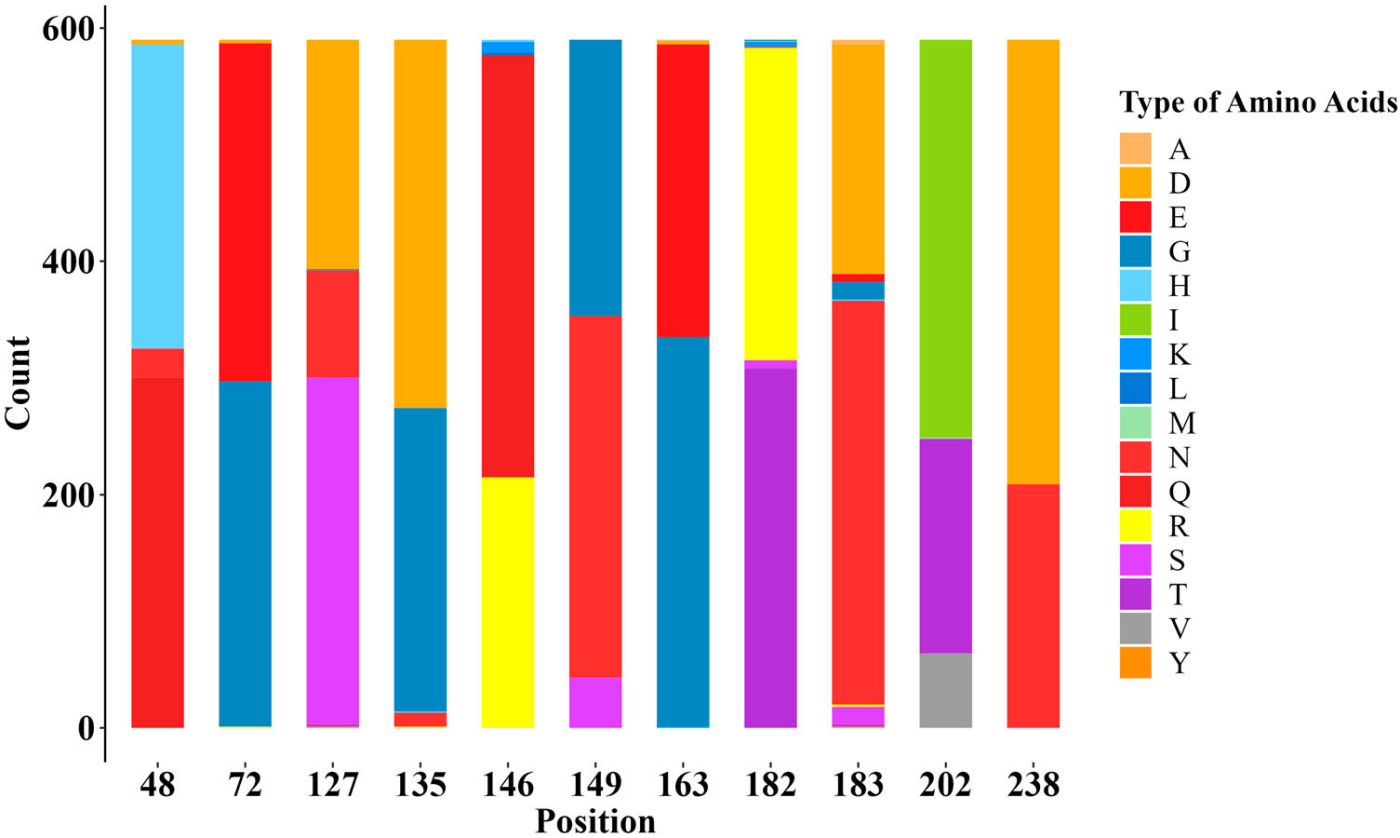
Stacked bar chart within 11 potential antigenic amino acid residues of 590 HA sequences from Clade 15 and Clade 16 strains. The y-axis (count) represents the counts of amino acids at each position in the sequence alignment. The colour of letter represents type of specific amino acid at this position.

Surface glycoprotein HA is a major antigen of influenza A virus, and amino acid substitutions in HA1 region can dramatically change antigenicity [42]. Based on the phylogenetic and antigenic relations between clade15 and clade16, the screening was performed to identify the difference in the proportion of each amino acid site in HA1 region between the two clades. 11 potential antigenic amino acid residues were found to be significantly different in clade 16 compared with clade15, including 48,72,127,135,146,149,163,182,183,202 and 238 (H9 numbering on HA). The detailed results are shown in Figures 4 and 5 and Supplementary Materials (Supplementary Table S6 and Figure S2).

## Discussion

Currently, the principal strategy for H9N2 prevention in poultry is the application of inactivated vaccines, while mutations in antigenic sites can result in vaccine failure. Epidemiological surveillance is a good way to capture these mutations. We conducted monitoring programs of H9N2 viruses in Eastern China from 2010 to 2020 and found a new designated clade 16 emerged in April of 2012. Major switches to antigenic properties were observed over the emergence of clade 16, with antigenic similarities between phylogenetically related strains.

Although being classified as LPAIV, H9N2 can also cause immunosuppression [43,44] as well as decreased egg production in the poultry industry, and cause significant morbidity and mortality in chickens while co-infection with other subtypes of AIV and bacteria [4]. Notably, Co-infections of H9N2 and other subtypes of AIV highlight the possibility of emergence of novel viral reassortment with unanticipated phenotypes [45–47]. To reduce the massive economic losses caused by the H9N2 viruses to the poultry industry, vaccination has been the principal strategy since 1998 in China [48,49]. However, the effectiveness of vaccines has always been challenged by antigenic drift because of the rapid mutations of RNA viruses. To update vaccine strains in time and prevent the transmission of H9N2 in poultry, it is very important to monitor the isolation rate and antigenic changes of H9N2 viruses.

Following inactivated vaccines such as F/98 applied in farms in China, sporadic outbreaks of H9N2 viruses persisted in immunized flocks. In 2010, a nationwide outbreak of H9N2 viruses suggested that H9N2 successfully obtained immune escape mutants, and the prevalence of H9N2 provided an opportunity for donating internal segments to reassort with other LPAIVs, leading to the emergence of H7N9 LPAIVs in 2013 [50,51].. We conducted monitoring programs of H9N2 viruses in Eastern China from 2010 to 2020, and approximately 900 samples were collected annually from chickens, ducks and geese in LBM (Figure S3A). The isolation rate of H9N2 viruses showed an overall increasing trend. The significant increase of H9N2 isolation rate in 2016 was associated with its prevalence in chickens, when 234 H9N2 were isolated from chickens in 252 total isolates and the decrease in 2020 was due to the outbreak of SARS-CoV-2, which affected our epidemiological investigation work. Although the data from online databases can’t provide a comprehensive representation of the results of epidemiological investigations, it can still offer insight into certain situations. Simple descriptive statistical analysis of the HA sequences number of H9N2 subtype AIVs uploaded to online databases for each year from 2010 to 2020 **(**Figure S3B**)** also indicated this increasing trend. A study of AIV epidemiological investigation in China from 2014 to 2016 discovered that H9N2 subtype AIV was one of the most prevalent subtypes [8]. Notably, compared to the statistic from 2014 to 2016, the isolation rate of H9N2 grew from 2016 to 2019, whereas other dominant subtypes such as H5N6 and H6N6 showed a decreased trend in the proportion of isolation rate [9]. In the meantime, H9N2 has become the dominant subtype in both chickens and ducks across China, replacing the dominant subtype H5N6 in ducks from 2014 to 2016, which further complicated the epidemiological dynamics [9]. These evidences revealed that H9N2 are still prevalent in China, serving as an alarm of the potential failure of the H9N2 vaccination [52], making it imperative to conduct further research on the antigenic characteristics.

The antigenic drift of H9N2 is mainly due to point mutations in the HA gene, which revealed the relations between genetic and antigenic [49]. The mutations in the HA1 domain lead to the antigenic drift [41]. Due to antigen variation of H9N2 subtype AIV, distinct antigenic groups formed, leading to the low immunity efficacy of the used vaccine strains, giving rise to the widespread circulation of H9N2 in China. Much work has focused on antigenic drift and associated molecular markers [17,49,53]. Phylogenetic analysis of the HA gene of H9N2 viruses is usually conducted to find key amino acid mutations, and antigenic analysis of the prevalent clades is more relevant. However, systematic studies of H9N2 genetic and antigenic have not been updated for a long time. There is currently a lack of universally accepted guidelines for H9N2 clade classification.Li et al. divided HA clades of H9N2 viruses into 16 clades (0-15) by referring to the nomenclature system for H5N1 viruses, and clades with median root-to-tip distances equal to or less than 20% of the median root-to-tip distance of all clades in the tree were selected as clusters [28].We updated the H9N2 clades based on this research work and found a new designated clade 16emerged in April of 2012, showing a difference in antigenic to clade15 according to the antigenic analysis.

The proportion of isolated strains from clade15 and clade16 showed that clade16 co-circulate with clade15 after 2013, and gradually displaced clade15 as the most prevalent clade. Unfortunately, only a small number of sequences after 2018 were downloaded from online databases, where the strict management policy for the live poultry markets as well as the interruption of epidemiological investigation work could be reasons, so the proportion of two clades in 2019 and 2020 may not be entirely accurate. Furthermore, unlike the phylogenetic trees illustrating the evolution of the HA gene of H3N2 subtype AIVs have a “cactus-like” shape with a strong temporal structure, the phylogenetic trees of the HA gene of H9N2 viruses have a tree shape controlled by non-selective population dynamic processes, which means the immune system selection pressure is absent or weak [54]. It is consistent with the fact that inactivated vaccines do not prevent H9N2 viruses from transmitting in chickens [52]. The strains from clade16 were genetically distinct from clade15, and considering the relations between genetic and antigenic, the change in genetic may have an effect on antigenicity, which contributed to the vaccine failure partly.

Antigenic analysis showed that major switches to antigenic properties had emerged over the emergence of clade 16. The antigenicity of the strain can be affected by a single mutation in the HA protein [55,56]. Therefore, it is essential to identify the molecular marker of this antigenic drift in the new clade.

Following that, amino acid residues with significant differences of HA1 between clade15 and clade16 HA1 were identified, and potential antigenic-related amino acid mutations were H48Q, G72E, S127D, D135G, Q146R, N149G, E163G, T182R, N183D, T202I, N238D (H9 numbering on HA). Our approach to identifying molecular markers causing antigenic drift is simpler and less time-consuming than the traditional method of identifying antigenic-associated molecular markers by single point mutations through the construction of plasmids and reverse genetics. Some of our identified molecular markers have been verified by experiments in several recent papers, which is sufficient to prove the accuracy of this approach [26,48]. Secondly, the strains we chose for antigenic analysis were naturally prevalent strains rather than theoretically existing strains. It is meaningful to analyze these strains from prevalent clades. Oppositely, our work based on bioinformatics mining for potential antigenic sites also has some limitations. Antigenic associated sites in small proportions may be missed and to ultimately validate the functionality of the identified amino acid mutations, bench work is still required to be done.

In conclusion, our work provides insight into the genetic basis and antigenic characterization of H9N2 subtype avian influenza virus from 2010 to 2020 in China and highlights the necessity of update of vaccine strains in time.

## Supplementary Material

Supplemental Material

Supplemental Material

Supplemental Material

Supplemental Material

Supplemental Material

Supplemental Material

Supplemental Material

Supplemental Material

Supplemental Material

## Data Availability

Accessions with relevant metadata are contained in Supplemental Tables S1 andS2, and the sequences are available on Gisaid. Results of HI tests are contained in Supplemental Table S3. Scripts for generating figures are available upon request.
