# Supplementary figures and images for: Emergence of a new designated clade 16 with significant antigenic drift in hemagglutinin gene of H9N2 subtype avian influenza virus in eastern China
Source: Emerg Microbes Infect. 2023 Aug 28;12(2):2249558. doi: 10.1080/22221751.2023.2249558 (PMC10467529; doi:10.1080/22221751.2023.2249558)

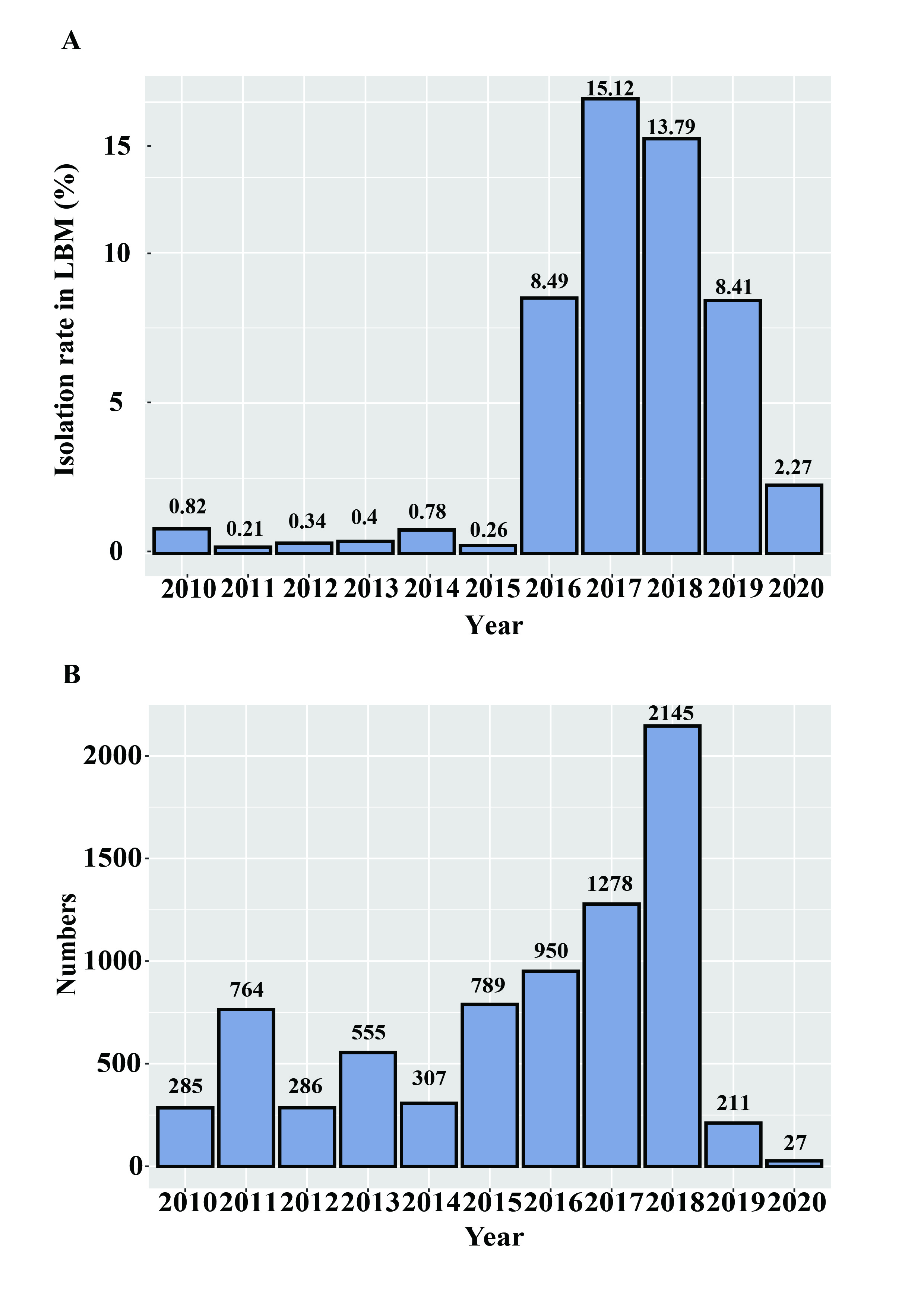

Supplement: Supplemental Material [file TEMI_A_2249558_SM1024.jpg]

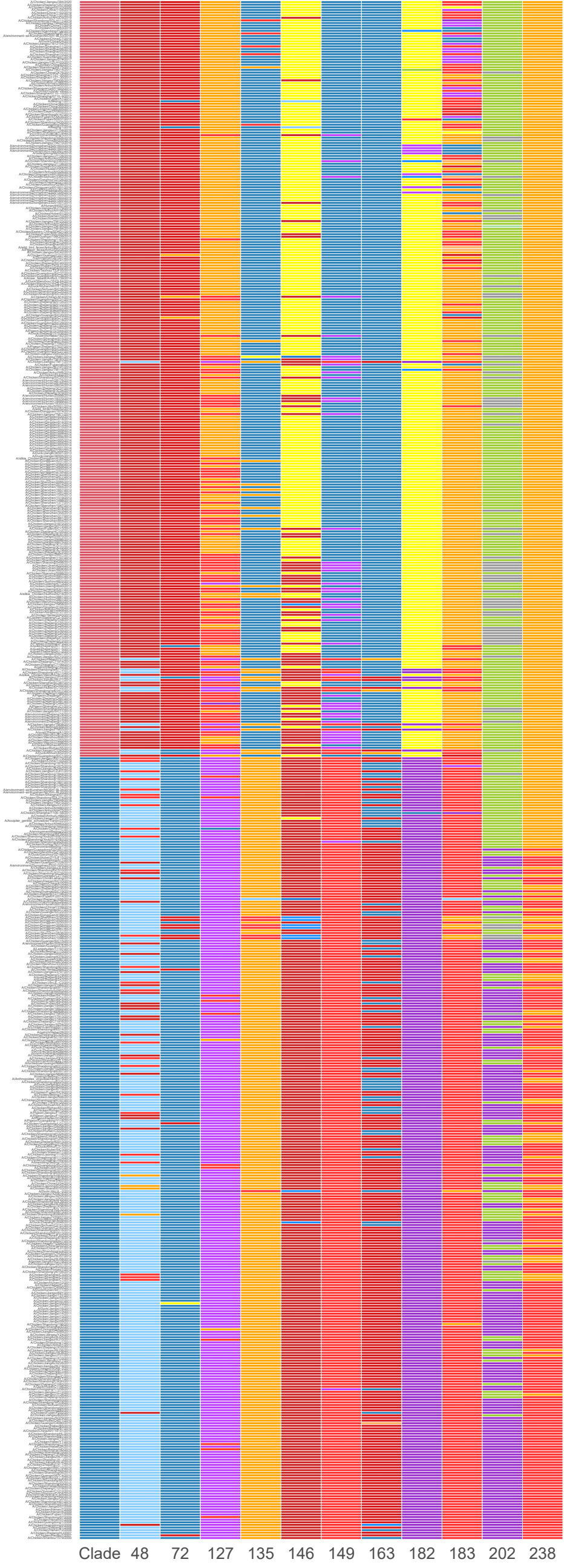

Clade 48 72 127 135 146 149 163 182 183 202 238

Supplement: Supplemental Material [file TEMI_A_2249558_SM0909.pdf]
