# Supplementary material for: Emergence of a new designated clade 16 with significant antigenic drift in hemagglutinin gene of H9N2 subtype avian influenza virus in eastern China
Source: Emerg Microbes Infect. 2023 Aug 28;12(2):2249558. doi: 10.1080/22221751.2023.2249558 (PMC10467529; doi:10.1080/22221751.2023.2249558)
Supplement: Supplemental Material [file TEMI_A_2249558_SM0827.pdf]

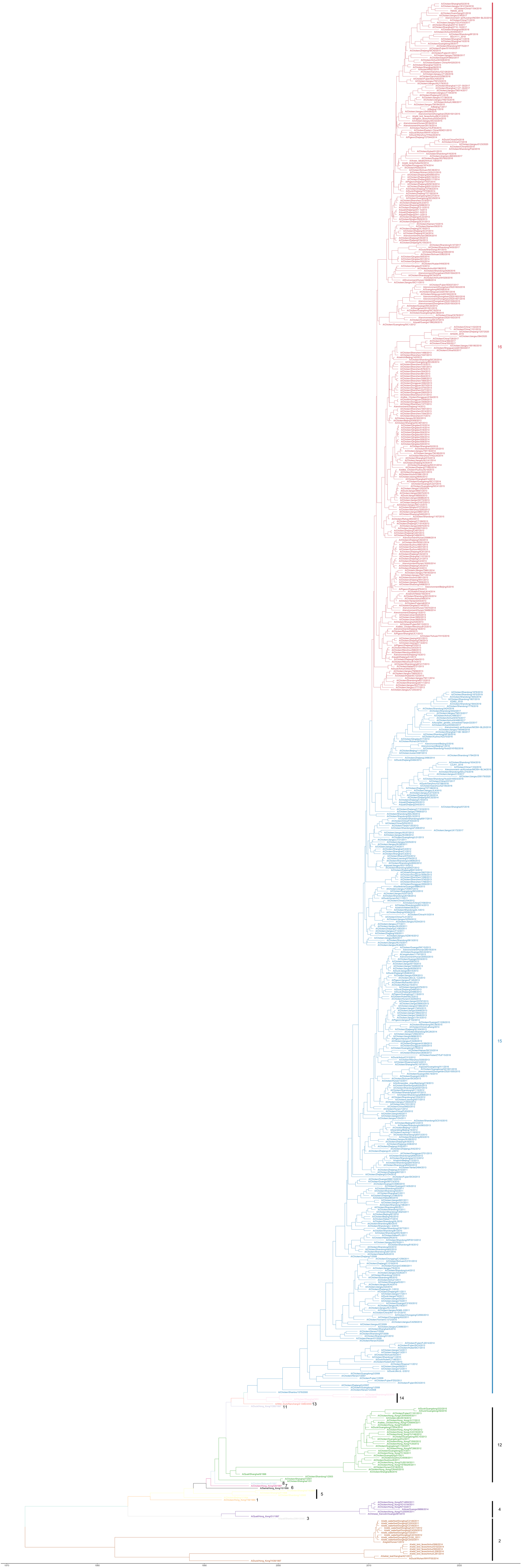
16
15
14
13
12
11
8
7
6
5
1
4
3
2
